# On Contagious Cerebro-spinal Meningitis of Horses1Centralblatt fur Bakteriologie, June, 1898, xxiii., p. 892. Translated for the Journal by Frederick D. Chester, Bacteriologist, Delaware College Agricultural Experiment Station, Newark, Delaware, October, 1898.

**Published:** 1898-12

**Authors:** 

**Affiliations:** Kiel


### ON CONTAGIOUS CEREBRO-SPINAL MENINGITIS OF HORSES.[1]

By Prof. Dr. Schneidemühl,

KIEL.

As cerebro-spinal meningitis of horses has recently made its appearance in many portions of Germany, and especially in the northern parts of the province of Saxony, it may be of interest to refer to certain recent investigations bearing upon the trouble. Our first knowledge of this disease, both as an affection of man

[1] Centralblatt fur Bakteriologie, June, 1898, xxiii., p. 892. Translated for the Journal by Frederick D. Chester, Bacteriologist, Delaware College Agricultural Experiment Station, Newark, Delaware, October, 1898.

and animals, dates from some time in the present century. Besides horses, the disease is also known to attack sheep, goats, cattle, and dogs.

According to Large, the trouble existed in Long Island between 1847 and 1849; and, according to Liautard, in a sporadic or epidemic form in 1861 in New York, New Jersey, and Pennsylvania. In Germany the disease was noted about the middle of 1860, and a great epidemic of the same occurred in Egypt in 1876.

During the past decade repeated losses, especially among horses, have occurred in North America, Egypt, Russia, England, France, Austro-Hungary, Saxony, in the Prussian Saxony provinces, and in Silesia. In the year 1895 cerebro-spinal meningitis became especially prevalent in the kingdom of Saxony, where it was investigated by Siedamgratzky and Schlegel.

Respecting the etiology of the disease, it was for a long time thought that the trouble was not carried from animal to animal, but was more of a miasmic origin. Siedamgratzky and Schlegel confirmed, by their observations of eight hundred cases, an earlier opinion that generally, although not exclusively, agricultural horses are more frequently attacked. No race of horses is, however, exempt; valuable coach-horses suffer in common with ordinary work-horses.

Although it is quite certain that there is some form of infecting virus causing the disease, nothing is yet known as to how this virus finds entrance; even the question of the transmission of the disease from animal to animal is not established. The exact period of incubation is also not known with any certainty, and may vary from weeks to months.

According to Siedamgratzky, the disease reaches its height during the winter and spring months, relaxing in summer; while, on the other hand, according to Sattler, from his observations in Southern New Jersey, the greater number of cases occurred during June and July, and with the approach of cold weather the trouble died out.

The disease appears to be less prevalent in the cities, where horses are more closely crowded than on farms, and hence the conclusion that the disease is not generally carried from animal to animal has some degree of logical support. On the other hand, it is claimed by Siedamgratzky that the specific infectious organism is capable of living, outside of the animal body, a saprophytic or facultative saprophytic existence, and by its multiplication under these conditions it may produce a miasma, which now and then will infect susceptible animals.

Up to the present time nothing has been known as to the distribution, propagation, and mode of entrance of this supposed organism. Concerning the latter some recent observations have been made.

Thomasse held to the analogy between purulent cerebro-spinal meningitis in man and in horses, and which is supposed to be due to a diplococcus or some other organism. Trambusti demonstrated in a case of cerebro-spinal meningitis in a sheep the *diplococcus pneumoniæ.*

Also, a buck-goat in the pathological laboratory of Florence, which had died of acute cerebro-spinal meningitis, was examined by the same author. In the same stall occupied by the latter was a she-goat, which also died later of the same disease. A child, which drank the milk of this last animal, also died with the same symptoms. An autopsy of the buck showed a sero-purulent infiltration of the meninges. In the exudate of the meninges and ventricles a diplococcus, united in chains of two, four, six, and eight elements, was found in considerable numbers. In the subpleural exudate, in the blood, and in the spleen they were much less abundant. The latter resembled Fraenkel's diplococcus, and stained in the ordinary way. Sections of the organ showed points of suppuration containing the diplococcus; also in the vessels of the pia mater the same organisms were found abundantly, either free or inclosed within the leucocytes. Also, in the vicinity of subpleural hemorrhagic areas, the diplococcus was found in groups. Cultures made from the cerebro-spinal exudate on agar or blood-serum appeared pure and showed, after twenty-four hours, small translucent, scarcely-visible colonies.

Subcutaneous injections of pure cultures, as well as of the heart-blood of a spontaneously dead goat, killed guinea-pigs and rabbits within twenty-four hours. In the peritoneal exudate and in the spleen the diplococcus was found in large numbers. From the above results Trambusti held to the view of an infectious cerebro-spinal meningitis whose virus was the same as that of the similar disease in man.

Siedamgratzky, in his bacteriological investigations, found a coccus, rarely a diplococcus, about 0.6 $\mu$ in diameter. It grew best at 38° C., and more slowly at the room-temperature, either in the presence or absence of air. It liquefied gelatin, was easily stained by the ordinary anilin colors, and not decolorized by Gram's method. The inoculation of the organism into horses showed in the case of intravenous injections no pathological effects analogous to the disease; but subdural injections brought about an acute meningitis and encephalitis, from which lesions the coccus was isolated pure. Said coccus failed to grow in fluid, but grew well on coagulated sera; it was therefore held that normal blood was not a favorable pabulum for the organism.

The easy and relatively rapid growth of the coccus on different media and at relatively low temperatures renders probable the view of Siedamgratzky that the organism in question has a wide distribution and longevity in stables, where it may also find favorable conditions for its multiplication. Furthermore, the apparent connection of the disease with suspicious feed might here find an explanation, since such food may form a suitable pabulum for the development of the germ in question.

Johne, during a recent outbreak of spinal meningitis in Saxony, made a number of bacteriological examinations. In the case of seven horses he isolated from the cerebro-spinal fluid a small diplococcus, about 0.4 to 0.8 $\mu$ in diameter, and in a single case he also found the same organism in the brain substance and in another in the blood. The organism stained easily with the ordinary anilin colors, but best with the Ziel carbol-fuchsin solution. Morphologically the diplococcus was characterized as the coffee-bean form; it occurred, however, now and then, as tetrads, and again as chains of two to six elements, with the line of fission of the diplococci coincident with the longer axis of the chains. In fresh material they were commonly surrounded by a capsule. Rarely was the organism found in the lymphoid or endothelial cells. This organism, according to Jager, as shown by preparations and cultures, seemed to be identical with the organism isolated in 1887 by Weichselbaum from cerebro-spinal meningitis in man, and named by him *diplococcus intracellularis.*

Inoculations made with the latter organism by Johne caused, in the case of intraperitoneal injections, the death of guinea-pigs in thirty-six hours with symptoms of toxæmia. Equally pathogenic was it in intraspinal injections into two goats. One of these died in thirty-six hours with symptoms of palsy, the other died after nine days with the typical picture of spinal meningitis. The autopsy showed in the last case fibro-purulent leptomeningitis, purulent ependymitis, and fibro-purulent spinal meningitis of the cervical cord. In the exudate in both cases the characteristic diplococcus was found in large numbers, as well as in the dorsal and lumbar cord.

A horse which was injected with this *diplococcus intracellularis* in the subdural space of the dorsal cord, in the region of the neck, became ill with typical clinical symptoms of apparently advanced cerebro-spinal meningitis. Three weeks after the above injection these symptoms had not disappeared. In the case of a second horse inoculated in like manner, but in which the greater part of the virus flowed back, and also in the case of another horse which had received an intravenous injection, there appeared in three to four days only slight cerebral symptoms.

Johne therefore claims that in the above diplococcus he has found the specific cause of cerebro-spinal meningitis in horses. Whether this last organism is identical with the ones discovered by Siedamgratzky and Schlegel further investigations must show.

With reference to the clinical symptoms, Siedamgratzky and Schlegel noted as constant a disturbance of the activity of the muscles of the neck and those concerned in chewing and swallowing; setting of the mouth; imperfect chewing; loss of the ability to seize hold of the feed; and, above all, loss of the power to effect suitable voluntary movements of the body.

With the disturbance of the activity of the muscles of the neck one notices a tonic and tense condition of the neck and an upward straining of the latter, the stag-neck (Hirschhals), simulating the conditions in tetanus. In endeavoring artificially to bend or move the neck the creature will frequently fall down and roll over.

As a rule, the disturbance in the powers of locomotion, as well as those of the coördination of the powers of equilibrium, are noticeable from the beginning. The animal stands with extended feet, and in walking staggers and often falls forward. It will then lie unconscious on the ground, making now and then spasmodic swimming movements.

The body temperature generally remains between 39° to 39.5° C. The pulse may be quite normal or somewhat accelerated.

The general symptoms are characterized by a more or less strong blunting of the senses, cramp, partial paralysis of the muscles of the head and neck, difficulty of movement, and disturbance of the equilibrium. The course of the disease is generally somewhat slow. The phenomena accelerate during the first week, when the trouble remains, with marked remission or disturbances, leading generally in the course of ten to eighteen days, with increasing palsy, to death.

Recovery takes place very slowly and is often followed by after-effects.

The mortality amounts, from the latest figures, to 76 to 80 per cent., and complete recovery takes place in only about 13 per cent. of the cases.

The anatomical picture, according to Siedamgratzky, shows a serous leptomeningitis which affects the brain and upper part of the cervical cord. In the brain the pia mater is strongly congested. Ramifying red areas occur at the base of the brain and in the medulla, disappearing backward.

As a rule, the cord in the vicinity of the second and third vertebræ is strongly reddened and now and then has a gelatinous consistency. The convolutions, especially near the base, are flattened and more or less filled with a serous fluid. In the white substance of the brain one often finds slight capillary hemorrhage, and some œdema. Most marked is the alteration of the pons and the medulla, extending to the region of the second and third vertebra. The appearances of the other organs show nothing characteristic.

Johne, in the observation of seven horses, found in the dead animals no lesion of cerebro-spinal meningitis or other inflammatory condition of the brain and spinal marrow, but only a venous engorgement with slight hemorrhage. The membranes of the brain appeared smooth and glistening, with only now and then a diffuse opacity of the latter. The anatomical changes showed rather the picture of a venous engorgement, but not a characteristic inflammatory hyperæmia. In all cases he noted in the spinal subdural and subarachnoid spaces, as well as in the ventricles of the brain, the presence of more or less (up to 150 c.c.) of a watery fluid, containing generally less than 1 per cent. of albumin. This, Johne considers to be a transudate and not a true exudate. Only in one case did he find in the brain substance and in the convolutions small reddish-yellow foci. Johne found in his examinations no anatomical lesions especially characteristic of the disease, death taking place from excessive weakness of the heart or by suffocation (Erstickung).

The author holds the disease to be due to a toxin which acts upon the central nervous system. The toxins he holds to be the specific products of the organism found in the subdural, subarachnoid spaces, and partly in the substance of the brain and spinal cord. The existing watery transudation he claims to be the result of the venous engorgement and of the effect of the toxins on the endothelium of the bloodvessels and lymphatics. Will not, therefore, the name cerebro-spinal meningitis from a comparative pathological standpoint have to fall? asks Johne. It is admitted that the disease in horses has clinically an evident similarity with the same disease in man, and also appears to stand etiologically in a certain relation to it, but from the standpoint of pathological anatomy it is not identical with it. In the disease in man there exists a specific inflammatory condition plus the toxæmia; in the case of horses only the toxæmia is evident.

To these statements of Johne it might be said that also in light cases in man the inflammatory lesions also fail; on the other hand, from the observations of others, it cannot be denied that acute cases of cerebro-spinal meningitis in horses are accompanied by evident inflammatory lesions of the brain and spinal cord.

Moreover, Johne himself shows how with pure cultures of the *diplococcus intracellularis equi* injected into goats he was able to produce lesions in the brain and spinal cord—*i.e.*, purulent lepto-meningitis and fibro-purulent meningitis which were quite similar to the same lesions in man.

---

Wounds of the Heart Not Always Fatal. A case was reported by Rehn, to the German Surgical Society, of a young man who was stabbed with a knife in the left chest. On the fourth day after the stabbing, the patient being moribund, an operation was decided upon. Incision was made into the fourth intercostal space and, the pericardium being exposed, a large clot was found in the pericardium, and an incised wound of 1-5 centimetre in length was found in the right ventricle. Three silk sutures were introduced into the opening, and the patient, after undergoing an attack of purulent pleurisy, made a good recovery. —*Therapeutic Notes*, P. D. & Co.

The possible extent of the spread of disease by the animals about us is still a matter of conjecture only. While it is more than probable that consumption and other diseases are distributed by domestic animals, good reason has been found for believing that the plague was introduced into Calcutta by rats, and, while mosquitoes probably inoculate the blood with disease germs, it now appears that flies may carry death in other ways. The last-named suggestion comes from Mid-Surrey. Two deaths from ptomaïn poisoning were attributed by the coroner's jury to eating lamb which was apparently sound and good, but which had been placed in a larder containing putrid tongue, and, as the two were not in proximity, the analyst believes that flies conveyed the poison from the tongue to the lamb. The obvious moral is that flies should be kept from larders and meat stores.

